# Production, extraction and characterization of *Chlorella vulgaris* soluble polysaccharides and their applications in AgNPs biosynthesis and biostimulation of plant growth

**DOI:** 10.1038/s41598-020-59945-w

**Published:** 2020-02-20

**Authors:** Noura El-Ahmady El-Naggar, Mervat H. Hussein, Sami A. Shaaban-Dessuuki, Shimaa R. Dalal

**Affiliations:** 1 Department of Bioprocess Development, Genetic Engineering and Biotechnology Research Institute, City of Scientific Research and Technological Applications, Alexandria, Egypt; 2 Botany Department, Faculty of Science, Mansoura University, Mansoura, Egypt

**Keywords:** Nanoparticles, Applied microbiology

## Abstract

*Chlorella vulgaris*, like a wide range of other microalgae, are able to grow mixotrophically. This maximizes its growth and production of polysaccharides (PS). The extracted polysaccharides have a complex monosaccharide composition (fructose, maltose, lactose and glucose), sulphate (210.65 ± 10.5 mg g^−1^ PS), uronic acids (171.97 ± 5.7 mg g^−1^ PS), total protein content (32.99 ± 2.1 mg g^−1^ PS), and total carbohydrate (495.44 ± 8.4 mg g^−1^ PS). Fourier Transform infrared spectroscopy (FT-IR) analysis of the extracted polysaccharides showed the presence of N–H, O–H, C–H, –CH_3_, >CH_2_, COO^−1^, S=O and the C=O functional groups. UV–Visible spectral analysis shows the presence of proteins, nucleic acids and chemical groups (ester, carbonyl, carboxyl and amine). Purified polysaccharides were light green in color and in a form of odorless powder. It was soluble in water but insoluble in other organic solvents. Thermogravimetric analysis demonstrates that *Chlorella vulgaris* soluble polysaccharide is thermostable until 240°C and degradation occurs in three distinct phases. Differential scanning calorimetry (DSC) analysis showed the characteristic exothermic transition of *Chlorella vulgaris* soluble polysaccharides with crystallization temperature peaks at 144.1°C, 162.3°C and 227.7°C. The X–ray diffractogram illustrated the semicrystalline nature of these polysaccharides. Silver nanoparticles (AgNPs) had been biosynthesized using a solution of *Chlorella vulgaris* soluble polysaccharides. The pale green color solution of soluble polysaccharides was turned brown when it was incubated for 24 hours with 100 mM silver nitrate in the dark, it showed peak maximum located at 430 nm. FT-IR analysis for the biosynthesized AgNPs reported the presence of carbonyl, –CH_3_, >CH_2_, C–H,–OH and –NH functional groups. Scanning and transmission electron microscopy show that AgNPs have spherical shape with an average particle size of 5.76. Energy-dispersive X-ray (EDX) analysis showed the dominance of silver. The biosynthesized silver nanoparticles were tested for its antimicrobial activity and have positive effects against *Bacillus* sp., *Erwinia* sp., *Candida* sp. Priming seeds of *Triticum vulgare* and *Phaseolus vulgaris* with polysaccharides solutions (3 and 5 mg mL^−1^) resulted in significant enhancement of seedling growth. Increased root length, leaf area, shoot length, photosynthetic pigments, protein content, carbohydrate content, fresh and dry biomass were observed, in addition these growth increments may be attributed to the increase of antioxidant activities.

## Introduction

Microalgae are some of the oldest, most economically promising organisms in the world^1^ and one of the richest sources of protein in addition to polysaccharides, carotenoids, phycobiliproteins, polysaccharides, vitamins and sterols^2^. Depending on the algal strain and growth conditions, algae can produce different valuable compounds, such as carbohydrates, proteins and lipids that serve as feedstocks for the production of biofuels^3^. *Chlorella* is one of the most cultivated eukaryotic green microalga, because it is extensively used in the pharmaceutical and beauty care products industry and as a health food and feed supplement^4^. *Chlorella vulgaris* contains 10% minerals and vitamins, 5% fiber, 20% carbohydrates, 20% fat, and 45% protein (w/w, dry basis)^5^.

Algal polysaccharides have many applications in agricultural, biomedical, and pharmaceutical fields^6^. Polysaccharides as natural biopolymers are easily modified, biocompatible, stable biodegradable, non-toxic and highly safe^7^ so they have a significant role in the drug delivery^8^. They are commercially applied as beverages, feed, food, emulsifiers, thickeners, stabilizers, etc.^9^. They also have biological activities, as being antitumor, antivirus, antihyperlipidemia, antioxidant and anticoagulant and established as a new drugs generation^8,10^. Extracellular polysaccharides produced by *Chlorella vulgaris* caused significant antitussive effects, anti-inflammatory and bronchodilatory in test animals. *Chlorella* extracellular polysaccharide seems to be an effective agent to prevent chronic inflammation of the airway, which is the predominant symptom of certain respiratory diseases, including bronchial asthma^11^. The polysaccharide content of microalgae biomass is gaining more importance as a feedstock for bioethanol production^12^. Microalgal polysaccharides have remarkable potential in the cosmetic industry as antioxidants for topical applications, including creams and lotions and hygroscopic agents^13^.

Silver nanoparticles (AgNPs) are commonly synthesized by laser ablation^14^, irradiation^15^, thermal treatment^16^ and chemical reduction^17^, which are energy-intensive, low yields, generate high levels of hazardous wastes, are difficult to scale up, and the use of organic solvents and toxic reducing agents may be required. Thus, these techniques yield extremely expensive materials. Moreover, the produced nanoparticles exhibit unwanted agglomeration with time. Hence, a green synthesis approach has been established to obtain comparatively cheap, safe, biocompatible, non-toxic, ecofriendly, size-controlled nanoparticles and easily scaled up for large-scale synthesis^18,19^. Biosynthesized silver nanoparticles are composites of inorganic and specific organic materials such as lipids, proteins or polysaccharides. These have unique physical and chemical properties that differ from the properties of traditionally produced nanoparticles^20^.

Silver nanoparticles biosynthesis has been successfully synthesized using many bacterial species^21^, fungi^22^, actinomycetes^23^, and plant extracts^24^. As well as, silver nanoparticles can also be biosynthesized by using β-D-glucose as a reducing agent and starch as a protective agent with gentle heat^25^.

The antimicrobial activities of AgNPs have been well documented and they are verified to possess antipermeability, antiangiogenic, antiviral, anti-inflammatory and antifungal activities^26^. AgNPs can be applied in medicine in order to minimize infections and avoid colonization of bacteria on protheses^27^, as antimicrobial agents to minimize the infections that occur during surgery in a surgically implanted catheters^28^, vascular grafts^29^, dental materials^30^, wound healing; bone stimulators^31^ and human skin and stainless steel materials^30^. Recently, silver as natural inorganic metal, non toxic and strong antibacterial agent, it is now used in various types of fabric fibers. AgNPs are also used on toilet seats, refrigerators, linings of washing machine, dishwashers and hygienic products including water treatment systems^32^.

Polysaccharides can serve as prebiotics (substances that stimulate the growth of beneficial bacteria in the digestive tract) and promote the growth of healthy gut microbiota^33^. Hernández-Herrera *et al*.^34^ reported that polysaccharides-enriched extracts from *Padina gymnospora* and *Ulva lactuca* can be used as an algal source to stimulate the growth of tomato (*Solanum lycopersicum* cv. Río Grande) at low-cost inorganic cultivation. Polysaccharides or oligosaccharides have increased root growth, developed higher yields, enhanced germination of seeds and increased pathogen resistance in different crops following the application of crude polysaccharides extracts on plants^35,36^.

The objectives of this study were to extract and characterize the soluble fraction of polysaccharides from the microchlorophyte *Chlorella vulgaris*, to synthesize silver nanoparticles using the extracted soluble polysaccharides, and to investigate the stimulatory effect of soluble polysaccharides on germination and seedling growth of both *Triticum vulgare* and *Phaseolus vulgaris* plants.

## Results and Discussion

Microalgae have the potential to grow under mixotrophic way of nutrition by integration of both the autotrophic and heterotrophic machineries by absorbing provided organic substrates in addition to atmospheric CO_2_ as a source of carbon. There are three modes of nutrition by which microalgae can grow autotrophy, hetrotrophy and mixotrophy. Glucose, mannitol, sodium acetate, maltose, and fructose were potential carbon substrates for growth enhancement of the mixotrophically grown fresh water microalga *Monoraphidium griffithii* NS16^37^. As reported by Wijffels and Barbosa^38^, biomass production of microalgae donates a variety of algal metabolites as enzymes, lipids, biopolymers, pigments, toxins, food supplements and renewable energy products as biodiesel and bioethanol. As had been found previously by Heredia-Arroyo *et al*.^39^, the microchlorophyte *Chlorella vulgaris* can be cultivated on some external carbon substrates as energy and carbon source for cell growth. Zhang *et al*.^40^ identified mixotrophy as being grown with organic carbon assimilation in light at the same time with carbon dioxide fixation.

### Extraction and characterization of *Chlorella vulgaris* soluble polysaccharides

The soluble polysaccharides extracted from *Chlorella vulgaris*. Ji *et al*.^41^ explained the rules of carbohydrates in algae; they reported that carbohydrates act in the cell walls as structural components and serves as inside cell-reserved compounds. The water-soluble fraction of *Chlorella vulgaris* polysaccharides yielded 174.46 mg g^−1^ dry biomass. According to Ji *et al*.^41^, carbohydrates are portioned in algal cells between cell walls as structural components and inside cell as reserved components.

### Chemical composition of extracted polysaccharides

The chemical composition of *Chlorella vulgaris* polysaccharides is given in the Table 1, showing total protein content (32.99 ± 2.1 mg g^−1^ PS), total carbohydrate content (495.44 ± 8.4 mg g^−1^ PS), sulfate content (210.65 ± 10.5 mg g^−1^ PS) and uronic acids (171.97 ± 5.7 mg g^−1^ PS). HPLC chromatogram revealed that the monosaccharide portion is composed of glucose major component and minor contents of fructose, maltose, lactose, rhamnose and arabinose. Conflict results had been reported about the chemical structure of *Chlorella* polysaccharides. Yim *et al*.^42^ documented that *Chlorella vulgaris* have homo-polysaccharides of galactose, moreover Nomoto *et al*.^43^ indicated that this homo-polysaccharides are composed of glucose, while the polysaccharides from other green microalgae are heteropolymers of glucose, xylose and galactose in various ratios. Fructose, fucose and rhamnose as well as uronic acids could be obtained. The present results of *Chlorella vulgaris* hetero-polysaccharides analysis revealed the presence of six monosaccharide units, fructose, glucose, maltose, lactose, rhamnose and arabinose in addition to other components as sulphate, uronic acids and protein. Such findings are in agreement with those obtained by Sui *et al*.^44^ who found that *Chlorella* sp. polysaccharides yield ranged from 13 to 19% and composed of six neutral sugars: glucose, galactose, mannose, xylose, arabinose and rhamnose. Our results are also consistent with those of Raposo *et al*.^2^, who studied polysaccharides extracted from both *Chlorella vulgaris* and *Porphyridium cruentum*. The main neutral sugar components of *Chlorella stigmatophora* are fructose, xylose and glucose^45^, while that of *Chlorella vulgaris* are rhamnose, arabinose, 2-O-methyl rhamnose and glucose^43^. In the same context Shi *et al*.^46^ reported that *Chlorella pyrenoidosa* polysaccharides is composed of galactose, glucose, mannose, xylose, arabinose and rhamnose. Furthermore, Sheng *et al*.^47^ stated that the monosaccharide composition of *Chlorella pyrenoidosa* was galactose, glucose, mannose, rhamnose and an unknown monosaccharide.

**Table 1:** Chemical composition of *Chlorella vulgaris* soluble polysaccharides (mg g^−1^).

Total protein	Total carbohydrates		Sulphate content		Uronic acid
32.993 ± 1.656	495.443 ± 4.497		210.654 ± 2.021		171.97 ± 1.861
**Monosaccharide composition (%)**					
**Fructose**	**Glucose**	**Maltose**	**Lactose**	**Rhamnose**	**Arabinose**
3.808	81.289	7.578	7.32	19.41	14.1

White and Barber^48^ documented that after acid hydrolysis of the purified polysaccharides from the cell walls of *Chlorella pyrenoidosa*, the following sugars were released: 8.5% glucuronic acid, galactose, mannose, xylose, arabinose and rhamnose in molar ratios 2.9: 1.0: 2.2: 2.7: 11.5.

### UV characterization of extracted *Chlorella vulgaris* soluble polysaccharides solution

**Figure 1:**
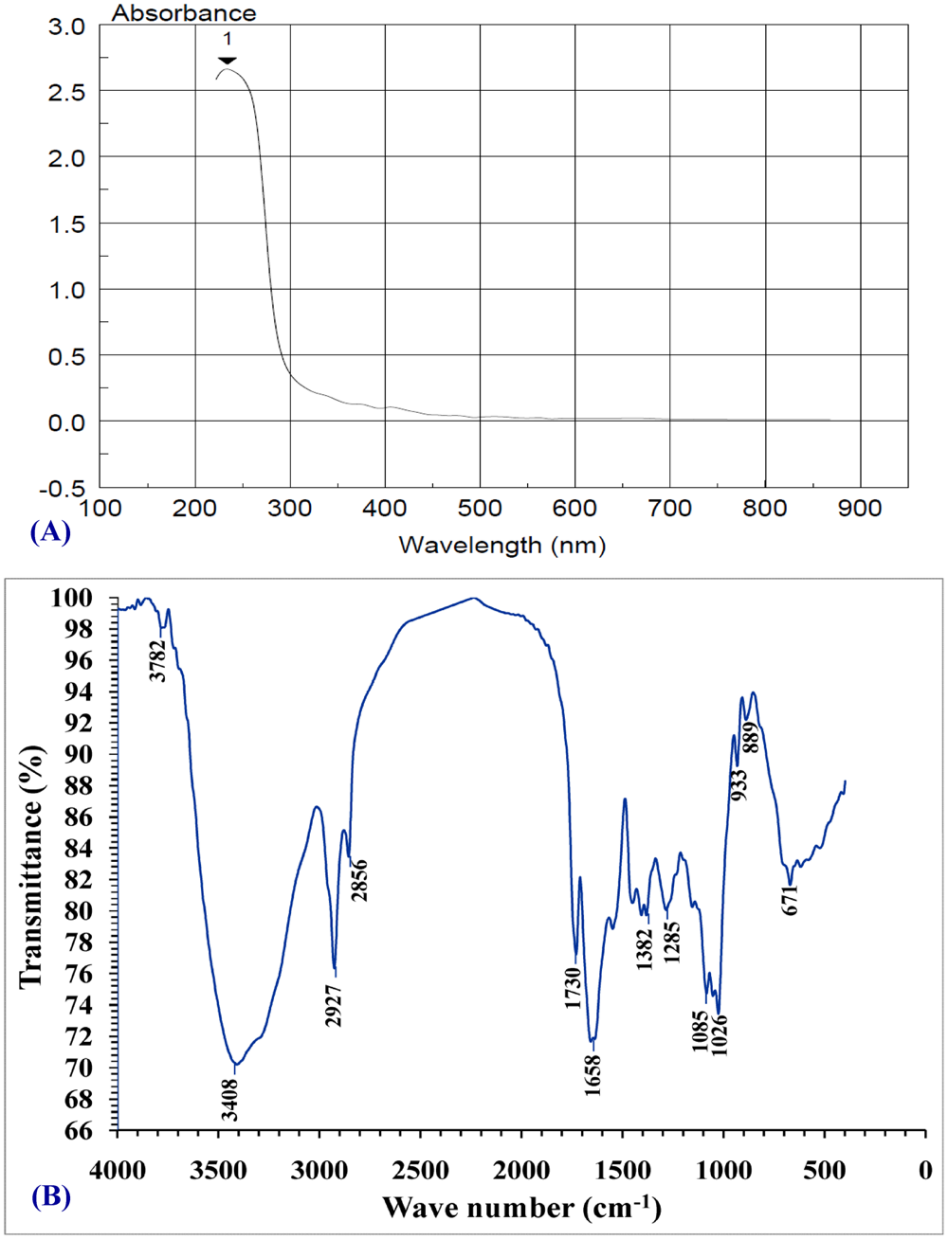
(**A**) UV – absorbance spectrum of aqueous solution of soluble polysaccharide extracted from *Chlorella vulgaris*, (**B**) FT-IR characterization of *Chloella vulgaris* soluble polysaccharide solution.

Figure 1A displays the ultraviolet scan spectrum of soluble polysaccharides, giving absorption peak at 234 nm. The ultra violet scan spectrum analysis of the soluble polysaccharides extracted from *Chlorella vulgaris* demonstrated the existence of proteins as well as nucleic acids due to the presence of absorption peak at 234 nm as reported by Jha *et al*.^49^. The ultra violet scan spectrum analysis revealed absorption peak within the 200–234 nm which is characteristic of chemical groups (ester, carbonyl, carboxyl and amine) as reported by Yun and Park^50^.

### Fourier transform infra red characterization of the extracted *Chlorella vulgaris* soluble polysaccharides

In order to further characterization and identification of the functional groups present in *Chlorella vulgaris* soluble polysaccharides structure, FT-IR analysis was performed. The FT-IR profile of polysaccharides, illustrated the characteristic functional chemical groups (Fig. 1B) showing peaks at 3782, 3408, 2927, 2856, 1730, 1658, 1382, 1285, 1085, 1026, 933, 889 and 671 cm^−1^.

FT-IR spectroscopy is an excellent tool to measure organic functional groups qualitatively especially C=O, N-H and O-H^51^.

The presence of peaks at 3782 and 3408 cm^−1^ are assigned to O–H stretching bonded and non bonded hydroxyl groups and water^52^. The peak at 2927 cm^−1^ corresponds to CH_2_ asymmetric stretching vibration^53^. The peak observed at 2856 cm^−1^ is attributed to C–H groups^54^, the peak spectrum at 2856 cm^−1^ corresponds to stretching of C–H, –CH_3_, and >CH_2_ functional groups^55^. Kamnev *et al*.^52^ demonstrated that, the band at 1730 cm^−1^ is related to the C=O stretching of aldehydes, ketones, carboxylic acids and esters. Generally, these results indicated that soluble polysaccharides extracted from *Chlorella vulgaris* contain uronic acids, sulfates and peptides, also Chakraborty *et al*.^56^ reported that *Chlorella vulgaris* polysaccharides comprises uronic acids, sulfates and peptides. Spectral peak around 1667 cm^−1^ corresponding to the absorbance of COO^−^ antisymmetric stretch^57^; absorption peak at 1658 cm^−1^ C–H stretching from the aromatic ring C–H stretching from the aromatic ring^58^. The spectrum shows the presence of a peak at 1382 cm^−1^ which is attributed to the O–H symmetrical stretching^59^. The absorption peak at 1285 cm^−1^ is attributed to C–O, bending vibration^60^ and the peak exists at 1085 cm^−1^ could be related to C–O stretching^61^ and also the peak exists at 1026 cm^−1^ was due to C–O stretch vibration^62^. Additionally, the peak at 933 cm^−1^ was assigned to the vibration of C–O–C ring deoxyribose^53^ and the peak observed at 889 cm^−1^ was attributed to the RR′CCH_2_ group^63^.

### Antioxidant activity of *Chlorella vulgaris* extracted polysaccharides (reducing power)

**Figure 2:**
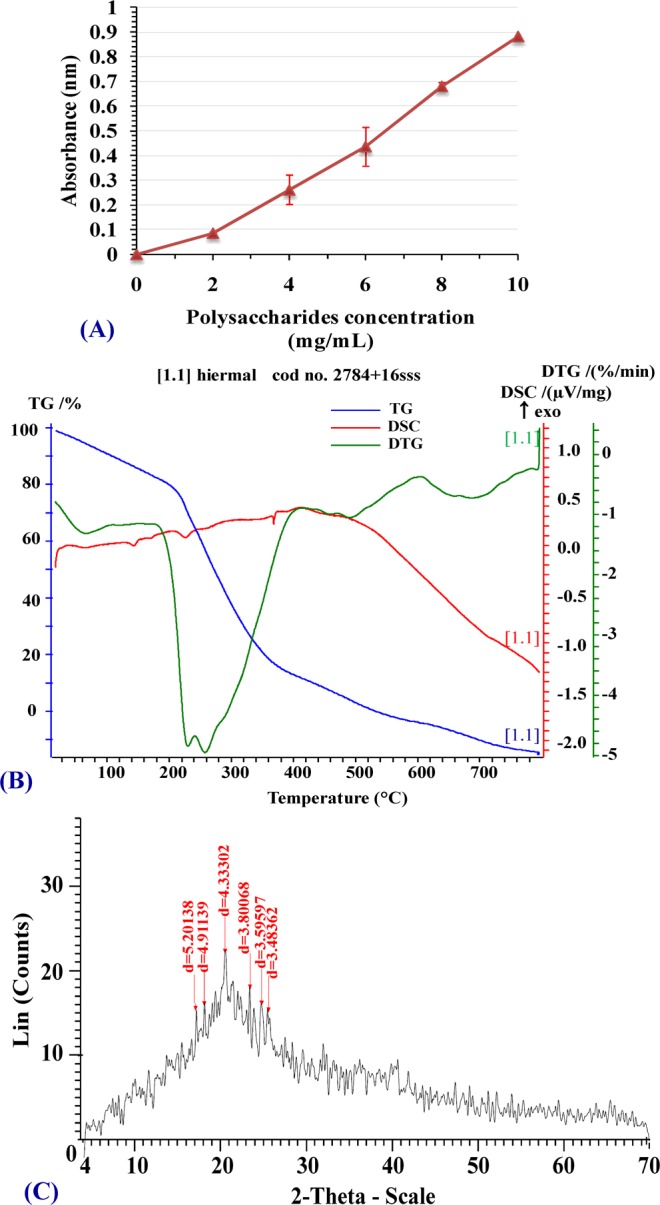
(**A**) Reducing capacity of *Chloella vulgaris* soluble polysaccharides. (**B**) Thermogravimetric analysis (TGA), DSC and (**C**) X-ray diffraction of extracted *Chloella vulgaris* soluble polysaccharides solution.

K_3_Fe(CN)_6_ reduction method was used to analyze the reducing capacity of the extracted polysaccharides. The results showed that with an increase in the concentration of the sample, the reduction increased (Fig. 2A). The reducing capacity of polysaccharides at a concentration of 10 mg mL^−1^ was 0.882 nm (absorbance at 700 nm).

Ferric reducing antioxidant potential method is a quantitative assay used for measuring the antioxidant potential of *Chlorella vulgaris* soluble polysaccharide. It is based on the reduction of ferricyanide [Fe (CN)_6]_^3−^ to ferro cyanide [Fe(CN)_6_]^4−^, which is a ferrous (Fe^2+^) derivative, in the presence of *Chlorella vulgaris* soluble polysaccharide. Then, the ferro cyanide [Fe(CN)_6_]^4−^ reacts with ferric chloride to form ferric-ferrous complex that is read colorimetrically with an absorption maximum at 700 nm. Wang *et al*.^64^ indicated that antioxidant activity has a direct, positive relationship with the reducing potential which depends on the type of sugar, molecular weight, the degree of sulfation and acetylation position and the glycosidic branching, consequently, the antioxidant potential of *Chlorella vulgaris* soluble polysaccharide can be attributed to sulfate fraction of the polysaccharides, contributing to the antioxidant properties. Qiao and Wang^65^ explained the positive relation between antioxidant activity and the reducing capacity.

Pulz and Gross^66^ indicated that microalgae were exposed to radical and oxidative stresses. The anti-oxidative scavenger complexes consequently defend their own cells against free radical oxidative stresses. Tannin-Spitz *et al*.^67^ reported that the soluble polysaccharides from *Porphyridium* sp. have shown antioxidant activity towards suppress oxidative damage to 3T3 cells and linoleic acid autooxidation, this bioactivity was dose-dependent. Duh *et al*.^68^ indicated that reducing capacity is a significant non-enzymatic anti-oxidant assays. Here the antioxidant power is based on the ability of ferric reduction in a redox-linked colourimetric reaction that includes single electron transfer. The results of Hussein *et al*.^69^ are in accordance with our results, reporting that the prominent absorbance value indicates stronger reducing potential, since the reducing power of ulvan samples increased with increasing concentration in dose-responding manner.

### Physical properties of extracted soluble polysaccharides

The extracted polysaccharides were pale green odorless powder. This polymer was insoluble in organic solvents and soluble in water, which conferred with general polysaccharide characteristics. The solution of polysaccharides was a clear with a pale green color, homogeneous liquid and after centrifugation there was no precipitation.

### Thermogravimetric analysis (TGA) of extracted polysaccharides

As indicated from the thermogram (Fig. 2B), *Chlorella vulgaris* soluble polysaccharides were thermally degraded in three identical steps. Step I, *Chlorella vulgaris* soluble polysaccharides lose 13.9% weight at elevating temperature from 25°C to 240°C this may be attributed to losing the physically bound water. High carboxy group levels in the *Chlorella vulgaris* soluble polysaccharides elevated the degradation of the 1^st^ phase (30–120°C) due to extra water molecules being attached to carboxyl group as described by Kumar *et al*.^70^. When the temperature rises above 240°C, the loss of weight increases until the temperature reached 420°C, when the polysaccharides beginning to decompose according to elimination of structure water (step II) with a 67.14% weight loss. Thermal characteristics of step III were recognized between 420°C to 654°C with 18.17% *Chlorella vulgaris* soluble polysaccharides weight loss that can be attributed to depolymerization conjugated with the breakage of C–C and C–O bonds in the ring structures leading to formation of CO, CO_2_ and H_2_O. Elevating temperature up to 800°C resulted in the formation of graphitic carbon and poly nuclear aromatic structures. It is concluded that *Chlorella vulgaris* soluble polysaccharides are thermostable until 240°C. The high thermostability of *Chlorella vulgaris* soluble polysaccharides may be due to the presence of uronic acids and sulfate groups that disallowed full polymer degradation. In addition, the thermogram is consistent with the hydrophilic nature of the polysaccharides functional groups^54,71^. The present thermogravimetric profile (TGA) data are in agreement with the thermal behavior of the soluble polysaccharide (ulvan) produced by *Ulva lactuca* and *Ulva fasciata* as demonstrated by Hussein *et al*.^69^.

### Differential scanning calorimetry (DSC) analysis of extracted polysaccharides

Analysis of DSC was performed to identify thermal transitions of the extracted *Chlorella* polysaccharides. Thermogram of DSC (Fig. 2B) showed the typical exothermic transition of the *Chlorella vulgaris* soluble polysaccharides biopolymer with crystallization temperature peak at 144.1°C (0.0053516 µv/mg) and the second crystallization temperature peak at 162.3°C (0.068322 µv/mg), the third crystallization temperature peak at 227.7°C (0.078219 µv/mg).

On elevating temperature, the viscosity of amorphous solid polymer decreased and at a definite temperature the particles become more free to move and be arranged into a crystalline state (crystallization temperature) as reported by Mishra *et al*.^72^. The conversion from amorphous solid polymer to crystalline solid polymer is an exothermic process, indicating the meltable semi–crystalline soluble polysaccharides as documented by Mishra *et al*.^72^. DSC is commonly used in pharmaceutical and polymer industries, recognizing polymer properties to illustrate their thermal transitions which can be used in characterization of materials as well as the thermal decomposition of polymeric materials. The specific temperature at which these transformations take place depends on the polymer’s structure^73^.

### X-ray diffraction of extracted polysaccharides

The XRD pattern revealed six strong peaks in the entire spectrum of 2θ values range between 4 and 70. These strong peaks have been detected at 2θ values of 17°, 18.5°, 21°, and 23.5°, 25°, 25.5° corresponding to 5.20, 4.91, 4.33, 3.80, 3.59 and 3.48 planes for polysacchride, respectively (Fig. 2C). The X–ray diffractogram of extracted *Chlorella vulgaris* polysaccharides illustrated the semicrystalline nature of these polysaccharides, as previously demonstrated by Alves *et al*.^71^.

### Rheological properties analysis of *Chlorella vulgaris* soluble polysaccharides

**Figure 3:**
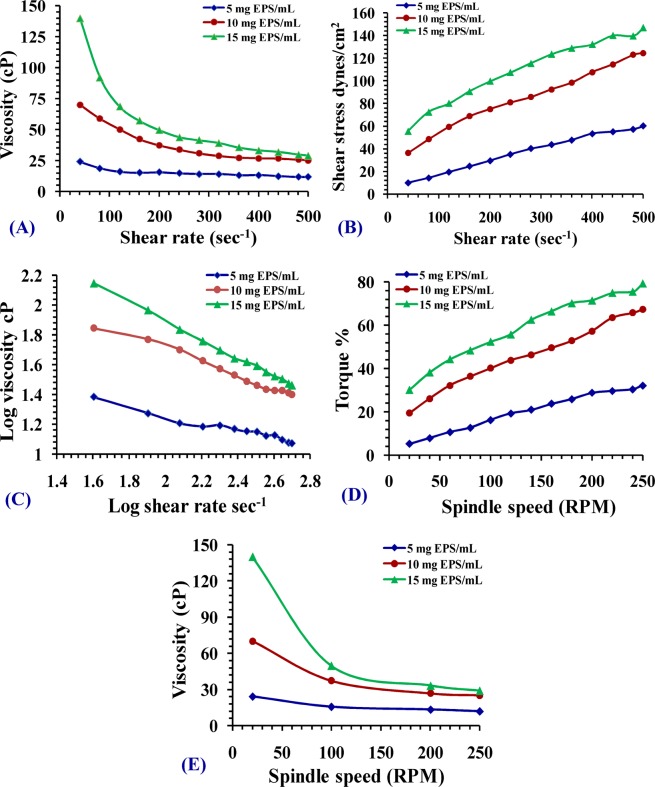
(**A**) Viscosity as a function of shear rate, (**B**) flow curve of the shear stress vs. shear rate, (**C**) log-log plot of the viscosity vs. shear rate, (**D**) rheogram of the Torque vs. spindle speed, (**E**) rheogram of the viscosity dependence of spindle speed (RPM) for aqueous solutions of *Chlorella vulgaris* PS at concentrations 5, 10 and 15 mg PS/mL.

The viscosity of the solutions of polysaccharides extracted from *Chlorella vulgaris* at a concentration of 5, 10 and 15 mg mL^−1^ as a meaning of shear rate (at a shear rate of 40 S^−1^) produced maximum values of 22.4, 73.1 and 120 centipoises (cP) viscosity; respectively (Fig. 3A). On the other hand, at elevating shear rate to 500 S^−1^, the solution viscosity declined to 11.5, 24.1 and 27.9 cP; respectively. Figure 3B illustrated the flow curves for soluble polysaccharides solutions with different concentrations. The shear stresses of soluble polysaccharides solutions decreased and estimate a restrictive stable value as the shear rate is reduced to zero at low shear rates, indicating that these soluble polysaccharides exhibit a limited yield stress. Obviously, higher yield stress values were obtained with higher polysaccharide levels. As illustrated in Fig. 3C, in aqueous solutions, soluble polysaccharides display a pseudoplastic or shear thinning characteristic. The uppermost viscosities and the notable shear thinning characteristics were reported to 15 mg mL^−1^ soluble polysaccharides, followed by 10 mg soluble polysaccharides mL^−1^ and finally 5 mg mL^−1^ soluble polysaccharides. Rheogram of Fig. 3D showed that the torque percent increases in increased spindle speed (RPM), while Fig. 3E demonstrated the relationship between viscosity of the solutions of soluble polysaccharides & spindle speed (RPM). It is evident that viscosity decreased with increased spindle speed.

Rising shear rate induced reduction in viscosity, while elevating *Chlorella vulgaris* polysaccharides concentrations caused increments in viscosity. This behavior exhibits distinctive non-Newtonian pseudo plastic pattern on shear thinning characterization in solutions as documented by Picout and Ross-Murphy^74^. This viscous performance was demonstrated by polysaccharides of other microalgae as reported by Bhatnagar *et al*.^75^. The dynamic viscosity behavior of soluble polysaccharides polymer is dependent on some variables such as polymer structure as well as its mass as reported by Freitas *et al*.^76^. Shear thinning pattern of the extracted soluble polysaccharides may be attributed to the hydrodynamic power resulted throughout the shear decline of soluble polysaccharides skeletal units as proposed by Khattar *et al*.^77^. Polysaccharide characterized by this viscous behavior is incorporated into food to alter rheological characteristics of the present water and thus change the product texture because of their ability to thicken or to cause gel formation as indicated by Sutherland^78^.

### Green synthesis of AgNPs by *Chloella vulgaris* soluble polysaccharides solution

**Figure 4:**
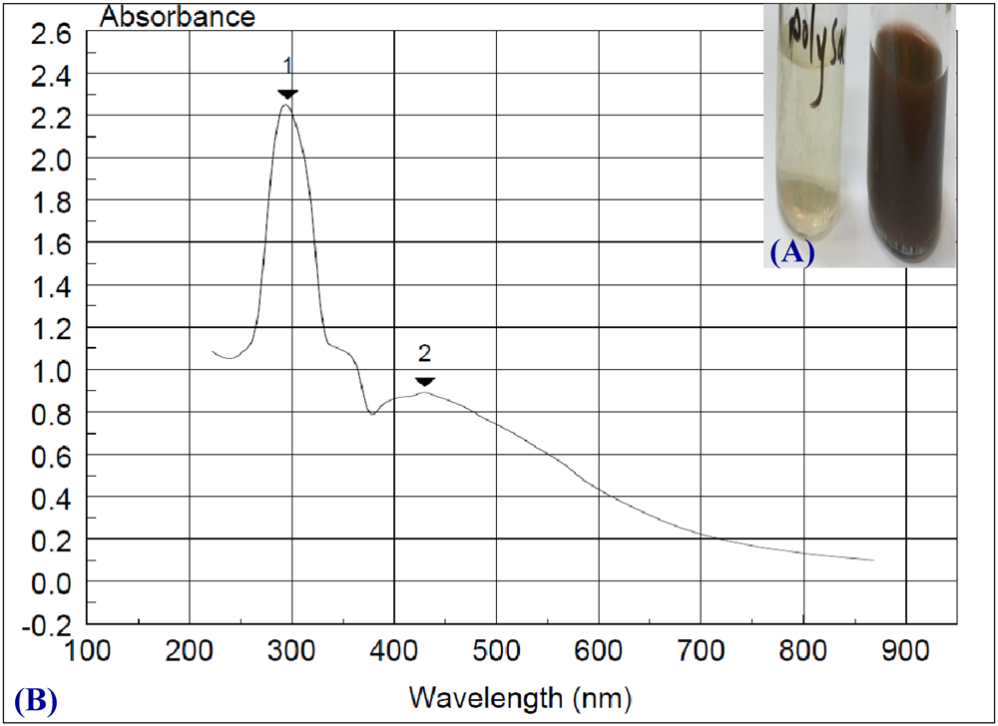
(**A**) Biosynthesized *Chloella vulgaris* soluble polysaccharide solution /silver nanoparticles and soluble polysaccharide control, (**B**) UV-absorbance spectrum of silver nanoparticles.

In the present study, AgNPs had been biologically synthesized using a solution of *Chlorella vulgaris* soluble polysaccharides. The pale green color solution of soluble polysaccharides produced by *Chlorella vulgaris* was turned brown when it was incubated for 24 hours with 100 mM silver nitrate in the dark to avoid the photolytic reaction (Fig. 4A). Phanjom and Ahmed^79^ reported that the dark-brown color generation can be attributed to the surface Plasmon resonance (SPR) excitation induced by silver atoms bioreduction and/or AgNPs^80^. The same finding has been established by Mohamedin *et al*.^81^ in the extracellular biosynthesis of silver nanoparticles by *Streptomyces aegyptia* NEAE102. The issue is shown as being eco–friendly and cost feasible instead of the conventional methods of silver nanoparticles biosynthesis. The described method is a rapid one and looked at a green approach for large scale protocol for AgNPs biosynthesis using polysaccharides solution that extracted from microalgae.

### UV absorbance of silver nanoparticles (AgNPs)

A complete synthesis of silver nanoparticles showed a gradual increase in absorbance with increasing the intensity of the yellow–brown color, it showed peak maximum located at 430 nm with absorbance value of 0.89 (Fig. 4B). The high color intensity (0.89) of the prepared nanoparticles solution at absorbance of 430 nm absorbance is attributed to a large number of nanoparticles produced in response to reduction power of the polysaccharides solution. As indicated from the results the Plasmon bands appear broad with an absorbance tail in the higher wavelengths. This can be attributed to size distribution of the nanoparticles, since this behavior demonstrated that the formed particles are polydispersed as explained by El–Rafie *et al*.^80^. Previous studies have shown that the biosynthesis of AgNPs by the bioreduction of silver into metallic silver may include DNA, sulfur–containing proteins^82,83^, and NADH and enzymes that depend on it^84^. Chun *et al*.^85^ indicated that NADH dependent enzymes may participate as an electron carrier in such biotransformation and biosynthetic reactions. The UV–Vis spectrum (Fig. 4B) corresponds, as previously mentioned; to AgNPs formation in the solution and the surface Plasmon resonance corresponding band is located at 430 nm. Ahmad *et al*.^22^ have shown that the surface Plasmon resonance band exists at 415 nm, and the particle size and dielectric constant of the medium affecting the exact location of the bands. In addition, the absence of any peaks at 335 and 560 nm, means that there are good stability as well as dispersion of the biosynthesized nanoparticles in the reaction mixture as postulated by Pandey *et al*.^86^.

### Fourier transform (FT–IR) characterization of the silver nanoparticles (AgNPs)

**Figure 5:**
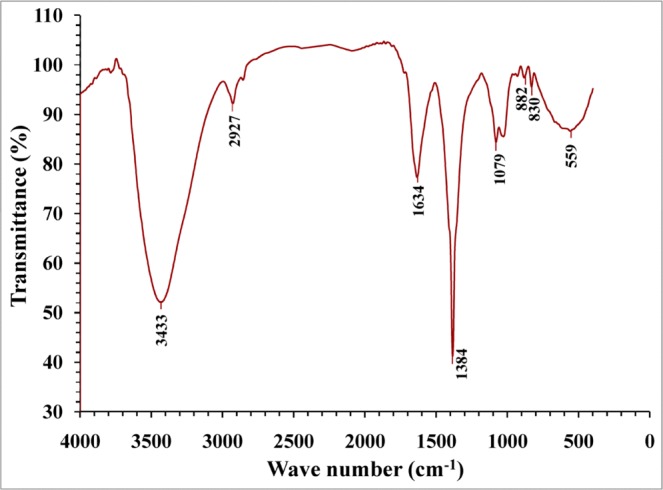
FT-IR characterization of silver nanoparticles.

FT–IR analysis was conducted to further characterization and identification of the functional groups found in the nanoparticles of silver. The FT–IR of nanoparticles, reveals characteristic functional groups (Fig. 5) showing peaks at 3433, 2927, 1634, 1384 cm^−1^, 1079, 882, 830, 559. FT–IR spectroscopy was applied in order to recognize and identify macromolecules responsible for the formation and bioreduction of silver cations and in addition to covering of the silver nanoparticles.

The band at 1384 cm^−1^ refers to deformation of >CH_2_, –CH_3_ and C–H groups; a minor peak at 2928 cm^−1^ corresponds to stretching of C–H group^77^. The peak at 3424 cm^−1^ may be corresponds to –OH or –NH groups and it is characteristic group to the algal polysaccharides^80^, whereas the spectral peaks at 1634 cm^−1^ refers to algal polysaccharide carbonyl groups and amide protein groups vibrations^55,80^. FT–IR data assigned to the presence of carbonyl groups, have great potential to join metal showing that proteins may constitute a layer in order to cover AgNPs to avoid agglomeration and hence the stabilization of the medium^87^. Moreover, data revealed the existence of reducing sugars in the polysaccharides solution which are able to induce a reduction in silver atom and synthesize the nanoparticles via biogenic routes^80^.

### Electron microscopy studies

**Figure 6:**
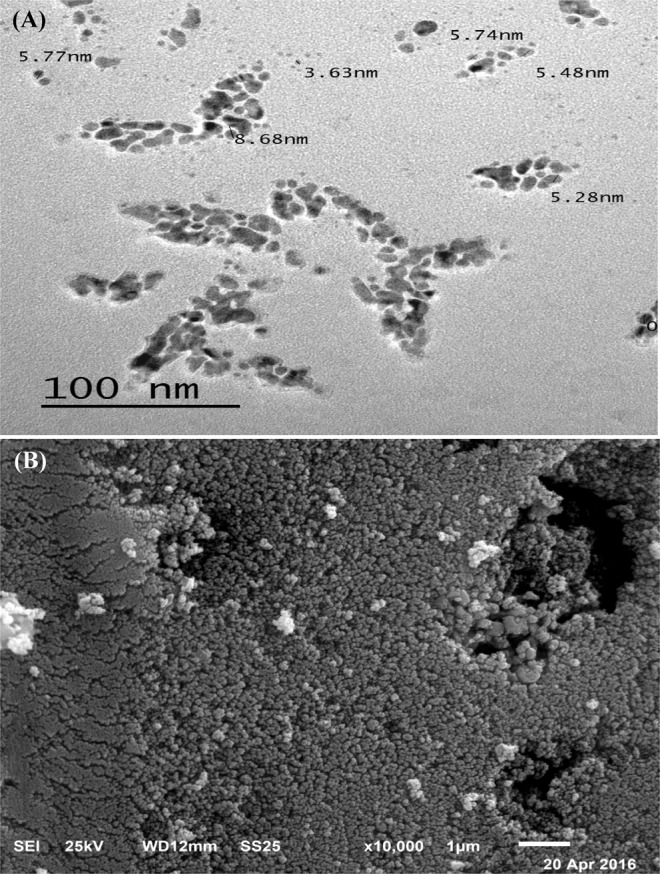
(**A**) TEM and (**B**) SEM electron micrograph picture of silver nanoparticles.

Transmission and scanning electron microscopy are effective methods for determining nanostructure morphology and size. Micrograph of TEM (transmission electron microscopy) of the biosynthesized silver nanoparticles showed the production of spherical nanoparticles with an average particle size (radius) of 5.76 nm and distribution range of 3.63–8.68 (Fig. 6A). Scanning electron microscopy illustrated the smooth surfaces of the biosynthesized silver nanoparticles (Fig. 6B). The findings of El-Naggar and Abdelwahed^88^ showed that the AgNPs are mainly spherical in their shape.

### Energy dispersive X–ray analysis (EDX) of silver nanoparticles

**Figure 7:**
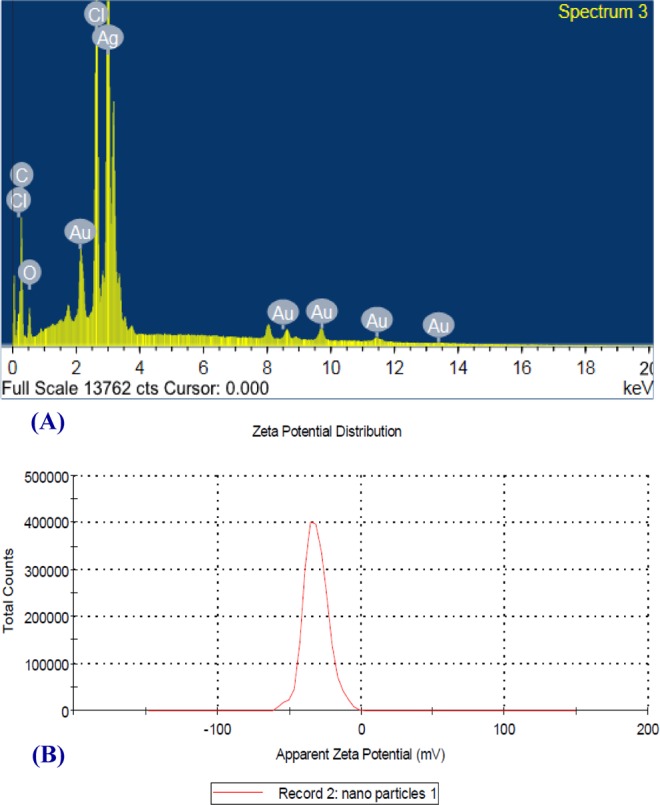
(**A**) Energy dispersive X-ray analysis (EDX) and (**B**) ZETA potential of silver nanoparticles.

Figure 7A shows standard EDX spectrum of the polysaccharides sample. The left part of the spectrum illustrated 3 peaks situated between 2 keV and 4 keV. Those maxima were characteristic to silver. The maximum peak located on the left side of the spectrum at approximately 0 to 1.7 keV is related to carbon, chlorine and oxygen. The hardly visible maxima located from 8–14 keV are characteristic of gold. In the analyzed sample, oxygen and carbon spots confirmed the existence of stabilizers consisting of alkyl chains. For quantitative analysis, the obtained EDX spectra were used, which showed high silver content (39.08%) in the examined sample. The optical absorption peak in the range between 3 and 4 keV is assigned to the metallic AgNPs absorption^89^. Except for silver, Fig. 7A demonstrated the presence of coal, gold and oxygen; the contents of them were 32.45%, 5.73% and 11.20%; respectively. However, according to the peaks of Au, C, O, and Cl, are attributing to emissions from the polysaccharides solution as suggested by Mukherjee *et al*.^90^.

### ZETA potential measurements

ZETA potential value for AgNPs (Fig. 7B) was found to be –31.3 mV; this reveals stability of the biosynthesized silver nanoparticles as reported by Hussein *et al*.^69^.

### Antimicrobial activity of biosynthesized AgNPs

**Figure 8:**
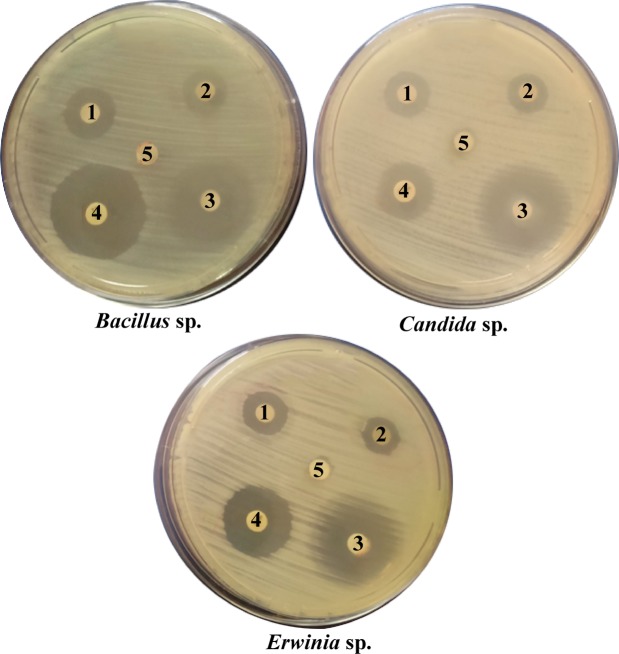
Effect of silver nanoparticles on *Bacillus* sp., *Erwinia* sp., *Candida* sp. (1- AgNPs 2- silver nitrate 3-streptomycin 4-tetracycline 5- penicillin).

Here is the antimicrobial potentiality of the biosynthesized AgNPs on 3 pathogenic microorganisms (Fig. 8) had been studied, the first was *Erwinia* which is a Gram negative bacteria, the second was *Bacillus* sp. which is a Gram positive bacteria, the third is *Candida* which belongs to the pathogenic fungi using specific antibiotics (pencillin 10 mg, tetracycline 30 mg and streptomycin 10 mg) as control. The synthesized AgNPs solutions had an inhibitory effect on all tested microorganisms, giving the following order *Bacillus*, *Erwinia*, *Candida* according to the radius of inhibition zone. The effect of nanoparticles on all tested organisms was more pronounced than that of silver nitrate and pencillin (Table 2). The most acceptable mechanism for explaining how silver nanoparticles affect antibacterial potentiality is through penetration of the cell wall of bacteria, and causes damage through interactions with compounds containing phosphorus and sulphur, including DNA^91^.

**Table 2:** Effect of silver nanoparticles on *Bacillus* sp., *Erwinia* sp. and *Candida* sp.

AgNPs and other antimicrobial agents	Microorganisms/inhibition zone diameter (mm)		
	*Bacillus* sp.	*Candida* sp.	*Erwinia* sp.
AgNPs	1.867 ± 0.087	1.6 ± 0.058	1.433 ± 0.022
AgNO_3_	1.533 ± 0.044	1.3 ± 0	1.233 ± 0.062
Penicillin	0 ± 0	0 ± 0	0 ± 0
Tetracycline	2.9 ± 0.033	2.066 ± 0.029	1.7 ± 0
Streptomycin	2.3 ± 0.058	2.7 ± 0.088	2.366 ± 0.073

### Promoting effect of *Chlorella vulgaris* polysaccharides on germination and seedling growth of both *Triticum vulgare* and *Phaseolus vulgaris* plants

The present results showed that priming seeds with (3, 5 mg mL^−1^ *Chlorella vulgaris* polysaccharides) significantly increased growth parameters (including dry and fresh weight, leaf area, shoot height and root length), photosynthetic pigments contents in seedlings leaves, carbohydrate, protein contents, and antioxidant activities in germinated seedlings of the *Phaseolus vulgaris* and *Triticum vulgare* compared to the control.

### Morphological criteria

**Figure 9:**
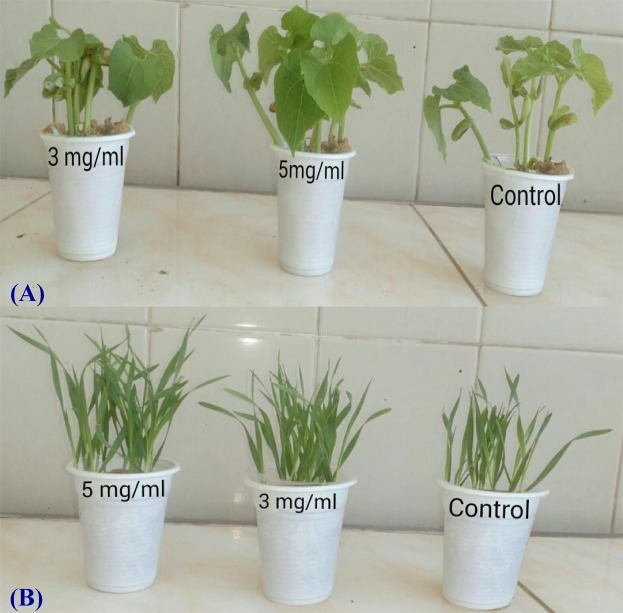
(**A**) Wheat (*Triticum vulgare*) growing seedlings and (**B**) *Phaseolus vulgaris* growing seedlings.

**Figure 10:**
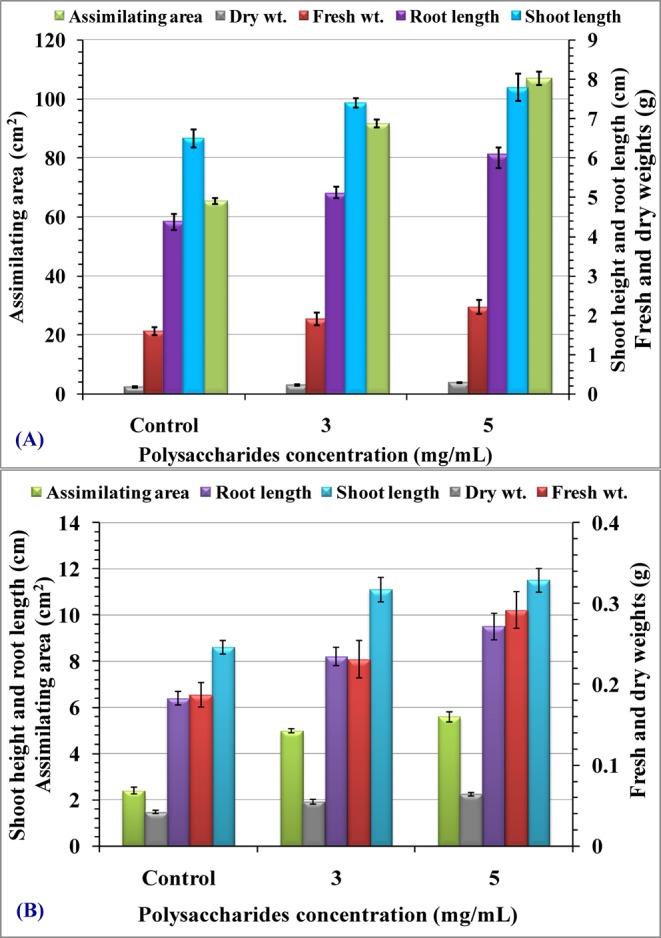
Priming effect of *Chlorella vulgaris* polysaccharides (3, 5 mg mL^−1^) on shoot height and root length (cm), assimilating area (cm^2^), fresh and dry weights (g) of *Triticum vulgare* (**A**) and *Phaseolus vulgaris* (**B**) seedlings after germination period of 10 days.

Figure 9 indicated enhancing priming effect of soluble polysaccharides solutions (3, 5 mg mL^−1^) on morphological criteria of both treated germinating seeds of *Triticum vulgare* and *Phaseolus vulgaris* when compared with control. There was a significant increase in shoot height, root length as well as assimilating area (first vegetative leaves) at the end of the period of growth (at 10 days) when compared with control. The induced effect on both investigated plants exhibit a concentration depending response. Soluble polysaccharides priming solutions stimulated significant increments in dry biomass for both tested plants and fresh biomass (in case of 5 mg mL^−1^ soluble polysaccharides priming solution) after a germination period of 10 days (Fig. 10).

### Metabolic criteria

**Figure 11:**
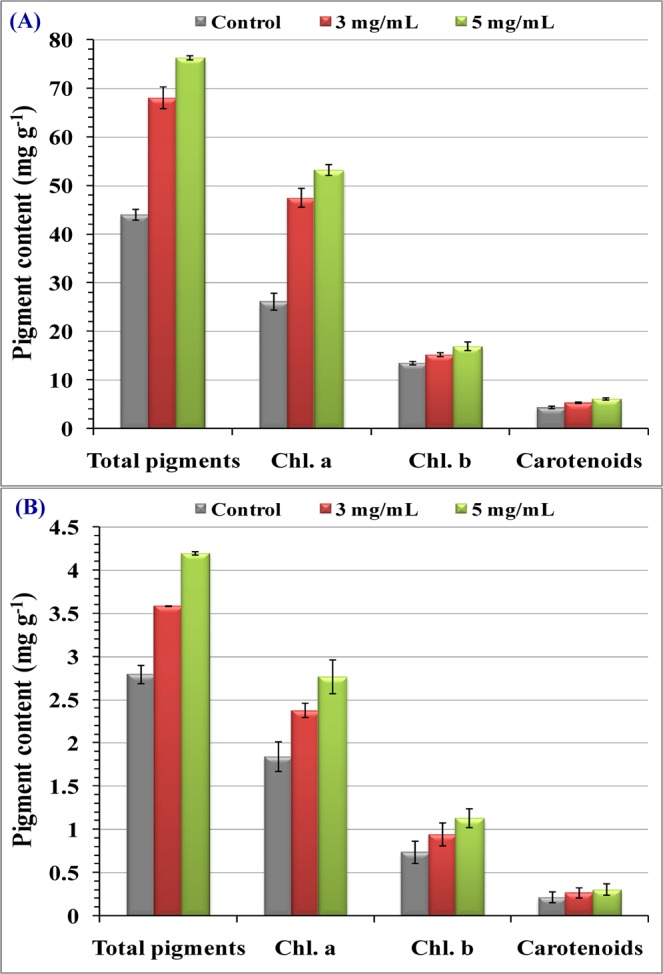
Priming effect of *Chlorella vulgaris* polysaccharides (3, 5 mg mL^−1^) on photosynthetic pigments content of *Triticum vulgare* (**A**) and *Phaseolus vulgaris* (**B**) after germination period (10 days).

Figure 11 illustrated that chlorophyll a, chlorophyll b and total pigments contents of treated seedling of *Triticum vulgare*, and chl b content of the *Phaseolus vulgaris* exhibit significant increments over control value in a dose response manner. While Chl b and carotenoids contents of *Phaseolus vulgaris* exhibit nonsignificant increments over control value. The effect of soluble polysaccharides on carbohydrate and protein contents of *Triticum vulgare* and *Phaeolus vigorous* seedlings is shown in Table 3. There are significant increments in both investigated plants. Guiacol peroxidase activity “GPX, EC: 1.11.1.7” induced significant increases in response to priming with different polysaccharides concentrations, giving the highest value in case of 5 mg mL^−1^ polysaccharides concentrations for both studied plants. Catalase activity “CAT, EC: 1.11.1.6”: polysaccharides priming induced significant positive responses in both studied plants with increasing polysaccharides concentration (Table 3). Total phenol contents of treated cultures showed significant increases above the control level in response to priming with different concentrations of polysaccharides for plants, *Triticum vulgaris* and *Phaseolus vulgare* as shown Table 3.

**Table 3:** Priming effect of *Chlorella vulgaris* polysaccharides (3, 5 mg mL^−1^) on protein content, carbohydrate content, enzymatic antioxidant activities and total phenolics of *Triticum vulgare* and *Phaseolus vulgaris* seedlings after germination period (10 days).

Polysaccharides	Total protein (mg g^−1^)	Total carbohydrates (mg g^−1^)	Catalase activity (U/mg)	Peroxidase activity (U/mg)	Total phenolics (mg g^−1^)
***Triticum vulgare***					
Control	29.251 ± 1.653	198.154 ± 2.888	19.271 ± 0.717	19.228 ± 0.535	0.021 ± 0.002
3 mg/mL	36.125 ± 1.696	219.178 ± 3.45	21.404 ± 0.728	21.525 ± 0.587	0.068 ± 0.002
5 mg/mL	47.132 ± 1.364	235.217 ± 2.833	30.62 ± 1.402	22.500 ± 0.929	0.117 ± 0.008
***Phaseolus vulgaris***					
Control	55.531 ± 1.966	351.213 ± 5.145	34.33 ± 0.454	22.947 ± 1.403	0.346 ± 0.02
3 mg/mL	76.124 ± 1.721	619.132 ± 2.314	45.08 ± 1.113	32.291 ± 0.702	0.413 ± 0.057
5 mg/mL	83.512 ± 3.112	637.132 ± 4.625	56.698 ± 2.04	35.814 ± 1.513	0.694 ± 0.01

The *Chlorella vulgaris* polysaccharides have the ability to furnish a microenvironment suitable for germination by buffering the osmotic disorders as well as stopping water loss^92^, in addition polysaccharides solutions provide high moisture for germination of the seeds as indicated by Vázquez *et al*.^93^. Present results revealed that priming with *Chlorella vulgaris* polysaccharides solutions promoted the vegetative growth of *Triticum vulgare* and *Phaseolus vulgaris* seedlings. This stimulating effect may be attributed to enhanced root growth, sportive ability, and antioxidant activities (enzymatic and non–enzymatic). In accordance with our results, plant growth stimulating activities as promoting root growth, sorptive ability, phosphorylation processes and nitrate assimilation capacity can stimulate seedling stem and tiller of several plants as indicated by Wen–yu^94^ and Osman *et al*.^95^. Cyanobacterial filtrates can stimulate synthesis of bioactive compounds that include regulators for plant growth, especially cytokinin, auxin and gibberellins^96,97^ in the germinated seeds which can increase the root length, shoot height and increases leaf chlorophyll content and increases of the leaf area as a result of chlorophyll accumulation in the leaves. The increase of leaf area, fresh and dry weight of studied seedlings as a result of increasing nutrients supplemented by soluble polysaccharides and their consumption^98^.

The supplied exogenous soluble polysaccharides are consumed as a source of carbon, the seedlings carbohydrate content can therefore be increased^99^ as a consequence of stimulation of pigment biosynthesis and the increase in the photosynthetic electron transport rate^100^. In addition to, the increased CO_2_ fixation^101^, the protein increase as a result of the increased N content^102^.

Priming of *Lupinus termis* seeds with different concentrations of soluble polysaccharides extracted from *Ulva* sp. led to significant increases in growth compared with control, in respect to shoot and root length, fresh and dry mass^69^. These findings were in perfect agreement with the results of Kavipriya *et al*.^103^, who stated that seaweed extracts stimulate *Vigna radiate* seed germination and growth parameters. Thirumaran *et al*.^104^ reported that seaweed extracts stimulate *Cyamopsis tetragonaloba* seed germination and growth parameters.

## Materials and Methods

### Isolation, culture conditions, purification and identification of algal isolates

*Chlorella vulgaris* were isolated from River Nile in Mansoura city. *Chlorella vulgaris* was grown in axenic cultures at 25 ± 2°C for 16 days incubation period under continuous illumination (3600 lux) in conical flasks of 500 mL volume, containing 200 mL BG11 medium^105^ and pH 7. Culture purification was according to Hoshaw and Rosowski^106^. Identification of *Chlorella vulgaris* was approved with the standard one according to Philipose^107^.

### Extraction and analysis of *Chlorella vulgaris* soluble polysaccharides

The extraction of polysaccharides was done according to hot water method^108^ with liquid/solid ratios of 1:20, at 70°C for 4 hours. The resulted extract was centrifuged; the residues were re–extracted 3 times. Soluble polysaccharides were precipitated using 90% ethanol in volume ratio 1 extract: 4 alcohol and left for 12 hours at 4°C and the precipitate was collected and dried at 60°C. Total carbohydrate content of extracted polysaccharides was estimated according to the method of Dubois *et al*.^109^. 0.1 mL of the extracted polysaccharides was raised up to 1 mL with distilled water and mixed well. 1 mL of 5% phenol solution and 5 mL of of concentrated sulfuric acid were added and carefully mixed well. The reaction mixture solution was placed in a water bath at 25°C for 20 min. The absorbance was measured spectrophotometrically at 490 nm. A blank tube was prepared with water instead of the extracted polysaccharides for each sample.

### *Chlorella vulgaris* soluble polysaccharides hydrolysis and composition analysis using (HPLC)

Chemical composition of the polysacchride was performed using a high performance liquid chromatography HPLC system^110^. After polysaccharides hydrolysis using 2 M trifluoroacetic acid at 100°C, monosaccharides analysis was performed by Agilent 1100 HPLC refractive index detector (RID) using a Hypersil ASP–2 column (4.6 × 250 mm) with a mobile phase of acetonitrile water (80: 20), a flow rate of 0.4 mL min^−1^. The column and optical unit temperatures were 35, 40°C; respectively. The mixed monosaccharides and hydrolyzate samples of the final extract were injected in the volume of 10 μL. Monosaccharides identification of the samples hydrolyzates was performed by comparing their obtained retention times with the standards for individual and combined six monosaccharides (xylose, fructose, sucrose, maltose, lactose and glucose) in the mobile phase in a concentration of 10 mg mL^−1^ under the same HPLC conditions.

### UV–Vis scan analysis of soluble polysaccharides solution

The UV–vis absorbance spectrum of an aqueous solution of the extracted soluble polysaccharides was recorded using ATI Unicam 5625 UV/VIS Vision Software V3.20 between 200 and 800 nm.

### Fourier transform infrared spectrometry (FT–IR) of soluble polysaccharides solution

FT–IR spectrum was performed using KBr on the Mattson 5000 FT–IR spectrometer. FT–IR spectra were measured in the frequency range from 4000 to 400 cm^−1^ ^111^.

### Estimation of total protein content of extracted soluble polysaccharide

According to the method of Lowry *et al*.^112^, the total protein content of the *Chlorella vulgaris* soluble polysaccharides was estimated.

### Antioxidant activity of extracted soluble polysaccharides (ferric reducing antioxidant power assay)

The antioxidant power reduction was calculated by the method described by Qiao *et al*.^113^. A series of test tubes have included the working standard of 0.2, 0.4, 0.6, 0.8 and 1 mL (10 mg/50 mL of soluble polysaccharides/distilled water) and with distilled water the volume was completed to 1 mL. Each test tube has been supplied with an additional 2.5 mL phosphate buffer (0.2 M, pH 6.6) and 2. 5 mL of 1%, w/v potassium ferricyanide [K_3_ Fe (CN)_6_] to reduce ferricyanide into ferrocyanide. The reaction mixture was incubated at a temperature of 50°C for 20 minutes. The reaction was stopped by adding 2.5 mL of 10% (w/v) trichloroacetic acid to the reaction mixture followed by centrifugation for 10 min at 3000 × *g*. 2.5 mL from the upper layer solution was mixed together with 2.5 mL distilled water and 0.5 mL of 0.1%, w/v FeCl_3_. The spectrophotometer was used to measure the absorbance at 700 nm against a blank sample. Blank was prepared same as sample without addition of soluble polysaccharides.

### Physical characterization of extracted polysaccharides

#### Thermogravimetric analysis (TGA) of the extracted polysaccharides

TGA was performed using a thermoanalyzer of the type 50–H. Previously, the sample of soluble polysaccharides was dried at 60°C for 1 hour and mounted in a platinum sample pan. Thermogravimetric analysis was performed in the range from 25 to 800°C, with a 20 mL min^−1^ flow rate, under a nitrogen atmosphere at increase of 10°C min^−1^. The chart was plotted as temperature versus weight loss (percentage).

#### Differential scanning colorimetry (DSC) analysis

A differential scan calorimeter (60–A) was used to estimate the polysaccharide pyrolysis pattern. Previously, the sample of soluble polysaccharides was dried at 60°C for 1 hour and mounted in an aluminum sample pan. The analysis was performed in a nitrogen atmosphere with a heating rate of 10°C min^−1^ and flow rate of 30 mL min^−1^. The thermogram was obtained between 25 to 250°C. The initial decomposition temperature for the test soluble polysaccharides, as is shown by TGA has been selected for the DSC upper limit. The chart was plotted as temperature versus heat flow.

#### X– ray diffraction (XRD)

The XRD analysis was performed using X–Pert Pro Diffractometer. Using the Debye–Scherrer formula, the crystalline domain size was determined.

#### Rheological analysis of soluble polysaccharides solution

BROOKFIELDDV-3 Ultra Programmable Rheometer^114^ was used for the measurement of rheological characteristics for 5, 10 and 15 mg mL^−1^ soluble polysaccharide solutions.

#### Green synthesis of silver nanoparticles (AgNPs) using *Chlorella vulgaris* soluble polysaccharides extract

Thirteen mg of *Chlorella vulgaris* soluble polysaccharides were dissolved in 90 mL distilled water and stirred at 70°C. Silver nitrate solution of 100 mM concentration was prepared. Then 3 mL AgNO_3_ were added drop by drop with continuous stirring, and then pH was adjusted to 10. The resulting solution was then centrifuged for three minutes at 3000 × *g*. Then the volume was raised to 100 mL using distilled water and continues stirring at 85°C for 20 minutes was done. After the reaction was completed, the solution was kept in the dark for 24 hours at room temperature (25°C), the solution color was changed to brown confirming the formation of AgNPs^80^.

### Characterization of biosynthesized silver nanoparticles by algal polysaccharides

#### UV–Visible spectral analysis

The formation of biosynthesized AgNPs has been certified visually by changes in color. Silver nanoparticles were characterized using Jenway UV/Visible– 2605 spectrophotometer, England. UV-Vis absorption spectrum was recorded by scanning the biosynthesized AgNPs in the range of 300–600 nm, at regular intervals. Control was polysaccharides solution free of silver nitrate^18^.

#### Fourier transform infra red (FTIR) spectroscopy analysis

FT–IR spectrum was performed for the lyophilized biosynthesized AgNPs sample to produce a pellet using using KBr (1:100). The FTIR spectrum was measured on Shimadzu FTIR–8400 S FTIR spectrometer in the range between 4000 and 500 cm^−1^ with 1 cm^−1^ of spectral resolution^19^.

#### Electron microscopic studies of AgNPs

Scanning electron microscopy examination of silver nanoparticles was carried out on JEOL JSM 6510/V, Japan, at the Electron Microscope Unit, Mansoura University. Transmission electron microscopy examination of silver nanoparticles was carried out on JEOL, JEM–2100, Japan, at the Electron Microscope Unit, Mansoura University.

#### Energy dispersive X–ray (EDX) spectroscopy analysis of AgNPs

EDX analysis of biosynthesized AgNPs was carried out to perform elemental analysis with JEOL JSM 6510/V, Japan, scanning electron microscope at Electron Microscope Unit, Mansoura University, Egypt, using an EDX detector operated at an accelerating voltage of 20 keV.

#### Zeta potential of AgNPs

Zeta potential value for AgNPs is determined using Malven Zeta size Nano–Zs90, Zeta potential analyzer.

#### Antimicrobial activity of AgNPs

The disc diffusion method^115^ was used for assessing the antimicrobial activity of AgNPs, silver nitrate, streptomycin, tetracycline and penicillin against *Bacillus* sp., *Erwinia* sp., *Candida* sp.

### Promoting effect of *Chlorella vulgaris* polysaccharides on germination and seedling growth of both *Triticum vulgare* and *Phaseolus vulgaris* plants

#### Plants and growth conditions

Two economic crop plants were chosen for this study as follows: Wheat (*Triticum vulgare*) and French bean (*Phaseolus vulgaris*). Seeds were supplied by the Ministry of Agriculture, Field Crop Institute, Agriculture Research Center, Giza, Egypt. They were examined for uniformity of size and shape before being surface sterilized by soaking for 3 minutes in 0.01% HgCl_2_ solution. Thereafter, they were thoroughly washed with running distilled water.

#### Experimental design

Seeds were rinsed for 15 minutes with sterile water. Then, they were soaked in priming solutions (3 mg and 5 mg polysaccharides mL^−1^) and other seeds were soaked in water (control) for 4 hours. Primed seeds were let to grow in plastic pots contain 300 g sandy soil for 10 days. After 10 days of growth, the samples of ten replicates were taken for determination of growth criteria, including root length, assimilating area, shoot height, fresh and dry weights and triplicate samples were taken for studying metabolism as determination of photosynthetic pigments, protein and carbohydrate contents^116,117^.

### Plant growth analysis

#### Morphological growth criteria

Both mean shoot height and mean Root lengths of 10 seedlings were estimated after germination period of 10 days. Mean leaf area (assimilating area) of 10 wheat seedlings was estimated usig the next equation of Quarrie and Jones^118^.

$${\rm{L}}{\rm{e}}{\rm{a}}{\rm{f}}\,{\rm{a}}{\rm{r}}{\rm{e}}{\rm{a}}\,{\rm{o}}{\rm{f}}\,Triticum\,vulgaris={\rm{l}}{\rm{e}}{\rm{n}}{\rm{g}}{\rm{t}}{\rm{h}}\times {\rm{b}}{\rm{r}}{\rm{e}}{\rm{a}}{\rm{d}}{\rm{t}}{\rm{h}}\times 0.75$$

For *Phaseolus vulgaris* mean leaf area of leaflets were estimated according to the following equation of Vermaat *et al*.^119^.

$${\rm{Assimilating}}\,{\rm{area}}=\pi (22/7)\times {\rm{leaf}}\,{\rm{radius}}\times {\rm{leaf}}\,{\rm{length}}.$$

Mean weight of 10 seedlings for each plant was recorded. Ten seedlings of each plant were dried at about 80°C in an oven until their weight was constant. Estimation of photosynthetic pigments^120^, total carbohydrate and total soluble sugars^109^ and protein content^112^ were recorded.

### Enzymatic antioxidant activity

#### Catalase activity

As described by Beers and Sizer^121^, catalase activity was determined by following the decline of the peroxides using spectrophotometric analysis at 240 nm. One catalase unit is defined as the amount of the enzyme that, under the stated conditions, decomposing one µmole of H_2_O_2_ at pH 7 and 25°C per minute, determined from the following equation:

One system breaks down one micromole of H_2_O_2_ per minute at 25°C and the pH 7.0.

$${\rm{Units}}/{\rm{mg}}=({\Delta {\rm{A}}}_{240}/min\ast 1000)/(43.6\ast {\rm{mg}}\,{\rm{enzyme}}/{\rm{mL}}\,{\rm{reaction}}\,{\rm{mixture}})$$

#### Peroxidase activity

Peroxidase activity was determined using the guaiacol oxidation method as described by Hakiman and Maziah^122^ (2009). The increase in absorbance due to the formation of tetraguaicol was measured at 470 nm. A unit of peroxidase activity was expressed as the change in absorbance per min.The specific activity as enzyme units per milligram of protein with extinction coefficient 6.39 mM^−1^ cm^−1^ refers to a unit of peroxidase activity.

#### Non enzymatic antioxidant activity (total phenolics determination)

Following a protocol modified from Gillespie *et al*.^123^, the total phenolic contents of the metabolic extracts were measured colorimetrically using Folin–Ciocalteu reagent. 20 µL of the ample and 40 µL of Folin–Ciocalteu reagent (25%) were added to a well of 96–well plate and incubated for a time of 5 min., then 140 µL of Na_2_CO_3_ solution (700 mM) was added to each well. After 2 hours incubation at room temperature, the resulting color was measured at 756 nm using the Spectra Max M5 reader and the resulting data were analyzed by softmax pro software. Triplicate analysis of all samples and standards were performed. Results are reported with the use of mg Gallic acid equivalents/g dry weight of *Chlorella vulgaris*, using Gallic acid as standard.

#### Statistical analysis

Results were analyzed according to Zobel *et al*.^124^ method using one–way analysis of variance, least significant difference (LSD) and values of *P* > 0.05 were considered statistically, non–significantly different, while those of *P* < 0.05 were statistically significantly different. Results were expressed as mean ± standard error.

## References

[1] Pittman JK, Dean AP, Osundeko O (2011). The potential of sustainable algal biofuel production using wastewater resources. Bioresource Technology.

[2] Raposo (2013). Bioactivity and applications of sulphated polysaccharides from marine microalgae. Marine Drugs.

[3] Skorupskaite V, Makareviciene V, Levisauskas D (2015). Optimization of mixotrophic cultivation of microalgae *Chlorella* sp. for biofuel production using response surface methodology. Algal Research.

[4] Sharma R, Singh GP, Sharma VK (2012). Effects of culture conditions on growth and biochemical profile of *Chlorella vulgaris*. Journal of Plant Pathology & Microbiology.

[5] Belasco W (1997). Algae burgers for a hungry world? The rise and fall of *Chlorella cuisine*. Technology and Culture.

[6] Liu Z, Jiao Y, Wang Y, Zhou C, Zhang Z (2008). Polysaccharides-based nanoparticles as drug delivery systems. Advanced Drug Delivery Reviews.

[7] Yang J, Han S, Zheng H, Dong H, Liu J (2015). Preparation and application of micro/nanoparticles based on natural polysaccharides. Carbohydrate Polymers.

[8] Wang SB, Chen AZ, Weng LJ, Chen MY, Xie XL (2004). Effect of drug-loading methods on drug load, encapsulation efficiency and release properties of alginate/poly-L-arginine/chitosan ternary complex microcapsules. Macromolecular Bioscience.

[9] Bixler HJ, Porse H (2011). A decade of change in the seaweed hydrocolloids industry. Journal of Applied Phycology.

[10] Song H (2018). Extraction optimization, purification, antioxidant activity, and preliminary structural characterization of crude polysaccharide from an arctic *Chlorella* sp. Polymers (Basel).

[11] Barboríková J (2019). Extracellular polysaccharide produced by *Chlorella vulgaris*–Chemical characterization and anti-asthmatic profile. International Journal of Biological Macromolecules.

[12] Lakatos GE (2019). Bioethanol production from microalgae polysaccharides. Folia Microbiologica.

[13] Barkia I, Saari N, Manning SR (2019). Microalgae for high-value products towards human health and nutrition. Marine Drugs.

[14] Tsuji T, Iryo K, Watanabe N, Tsuji M (2002). Preparation of silver nanoparticles by laser ablation in solution: influence of laser wavelength on particle size. Applied Surface Science.

[15] Shao K, Yao J-N (2006). Preparation of silver nanoparticles via a non-template method. Materials Letters.

[16] Sun X, Luo Y (2005). Preparation and size control of silver nanoparticles by a thermal method. Materials Letters.

[17] Peterson MS, Bouwman J, Chen A, Deutsch M (2007). Inorganic metallodielectric materials fabricated using two single-step methods based on the Tollen’s process. Journal of Colloid and Interface Science.

[18] El-Naggar NE, Hussein MH, El-Sawah AA (2017). Bio-fabrication of silver nanoparticles by phycocyanin, characterization, *in vitro* anticancer activity against breast cancer cell line and *in vivo* cytotxicity. Scientific Reports.

[19] El-Naggar NE, Hussein MH, El-Sawah AA (2018). Phycobiliprotein-mediated synthesis of biogenic silver nanoparticles, characterization, *in vitro* and *in vivo* assessment of anticancer activities. Scientific Reports.

[20] Tsibakhashvili NY (2011). Microbial synthesis of silver nanoparticles by *Streptomyces glaucus* and *Spirulina platensis*. Advanced Science Letters.

[21] Das R, Gang S, Nath SS (2011). Preparation and antibacterial activity of silver nanoparticles. Journal of Biomaterials and Nanobiotechnology.

[22] Ahmad A (2003). Extracellular biosynthesis of silver nanoparticles using the fungus *Fusarium oxysporum*. Colloids and surfaces B: Biointerfaces.

[23] El-Naggar NE, Abdelwahed NA, Darwesh OM (2014). Fabrication of biogenic antimicrobial silver nanoparticles by *Streptomyces aegyptia* NEAE 102 as eco-friendly nanofactory. Journal of Microbiology and Biotechnology.

[24] Masurkar SA, Chaudhari PR, Shidore VB, Kamble SP (2011). Rapid biosynthesis of silver nanoparticles using *Cymbopogan citratus* (lemongrass) and its antimicrobial activity. Nano-Micro Letters.

[25] Mochochoko T, Oluwafemi OS, Jumbam DN, Songca SP (2013). Green synthesis of silver nanoparticles using cellulose extracted from an aquatic weed; water hyacinth. Carbohydrate Polymers.

[26] Kalishwaralal K (2010). Biosynthesis of silver and gold nanoparticles using *Brevibacterium casei*. Colloids and surfaces B: Biointerfaces.

[27] Guzmán MG, Dille J, Godet S (2008). Synthesis of silver nanoparticles by chemical reduction method and their antibacterial activity. World Academy of Science, Engineering and Technology, International Journal of Chemical, Molecular, Nuclear, Materials and Metallurgical Engineering.

[28] Zhu Z, Kai L, Wang Y (2006). Synthesis and applications of hyperbranched polyesters—preparation and characterization of crystalline silver nanoparticles. Materials Chemistry and Physics.

[29] Edelstein, A. S. & Cammaratra, R. Nanomaterials: synthesis, properties and applications. (CRC Press, 1998).

[30] Maillard M, Giorgio S, Pileni M-P (2002). Silver nanodisks. Advanced Materials.

[31] Ibrahim FM, El-Hawary YM, Butler IS, Mostafa SI (2014). Bone repair stimulation in rat mandible by new chitosan silver (I) complexes. International Journal of Polymeric Materials and Polymeric Biomaterials.

[32] Rai M, Yadav A, Gade A (2009). Silver nanoparticles as a new generation of antimicrobials. Biotechnology Advances.

[33] Vidanarachchi J, Iji P, Mikkelsen L, Sims I, Choct M (2009). Isolation and characterization of water-soluble prebiotic compounds from Australian and New Zealand plants. Carbohydrate Polymers.

[34] Hernández-Herrera, R. M., Santacruz-Ruvalcaba, F., Zañudo-Hernández, J. & Hernández-Carmona, G. Activity of seaweed extracts and polysaccharide–enriched extracts from *Ulva lactuca* and *Padina gymnospora* as growth promoters of tomato and mung bean plants. *Journal of Applied Phycology*, 1–12 (2016).

[35] Laporte D (2007). Structurally unrelated oligosaccharides obtained from marine macroalgae differentially stimulate growth and defense against TMV in tobacco plants. Journal of Applied Phycology.

[36] Chandía N, Matsuhiro B (2008). Characterization of a fucoidan from *Lessonia vadosa* (Phaeophyta) and its anticoagulant and elicitor properties. International Journal of Biological Macromolecules.

[37] Yee W (2015). Feasibility of various carbon sources and plant materials in enhancing the growth and biomass productivity of the freshwater microalgae *Monoraphidium griffithii* NS16. Bioresource Technology.

[38] Wijffels R, Barbosa M (2010). An outlook on microalgal biofuels (vol 10, pg 67, 2008). Science.

[39] Heredia-Arroyo T, Wei W, Ruan R, Hu B (2011). Mixotrophic cultivation of *Chlorella vulgaris* and its potential application for the oil accumulation from non–sugar materials. Biomass and Bioenergy.

[40] Zhang X-W, Chen F, Johns MR (1999). Kinetic models for heterotrophic growth of *Chlamydomonas reinhardtii* in batch and fed–batch cultures. Process Biochemistry.

[41] Ji Y (2014). Mixotrophic growth and biochemical analysis of *Chlorella vulgaris* cultivated with diluted monosodium glutamate wastewater. Bioresource Technology.

[42] Yim JH, Kim SJ, Ahn SH, Lee HK (2007). Characterization of a novel bioflocculant, p–KG03, from a marine dinoflagellate, *Gyrodinium impudicum* KG03. Bioresource Technology.

[43] Nomoto K, Yokokura T, Satoh H, Mutai M (1983). Antitumor activity of *Chlorella* extract, PCM-4, by oral administration. Gan to kagakuryoho. Cancer & Chemotherapy.

[44] Sui Z, Gizaw Y, BeMiller JN (2012). Extraction of polysaccharides from a species of *Chlorella*. Carbohydrate Polymers.

[45] Guzman‐Murillo M, Ascencio F (2000). Anti-adhesive activity of sulphated exopolysaccharides of microalgae on attachment of red sore disease-associated bacteria and *Helicobacter pylori* to tissue culture cells. Letters in Applied Microbiology.

[46] Shi Y, Sheng J, Yang F, Hu Q (2007). Purification and identification of polysaccharide derived from *Chlorella pyrenoidosa*. Food Chemistry.

[47] Sheng J (2007). Preparation, identification and their antitumor activities *in vitro* of polysaccharides from *Chlorella pyrenoidosa*. Food Chemistry.

[48] White R, Barber G (1972). An acidic polysaccharide from the cell wall of *Chlorella pyrenoidosa*. Biochimica et Biophysica Acta (BBA)–General Subjects.

[49] Jha M, Venkataraman G, Kaushik B (1987). Response of *Westiellopsis prolifica* and *Anabaena* sp. to salt stress. MIRCEN journal of Applied Microbiology and Biotechnology.

[50] Yun U, Park H (2003). Physical properties of an extracellular polysaccharide produced by *Bacillus* sp. CP912. Letters in Applied Microbiology.

[51] Qian J-Y, Chen W, Zhang W-M, Zhang H (2009). Adulteration identification of some fungal polysaccharides with SEM, XRD, IR and optical rotation: A primary approach. Carbohydrate Polymers.

[52] Kamnev AA (2002). Fourier transform infrared spectroscopic characterization of heavy metal-induced metabolic changes in the plant-associated soil bacterium *Azospirillum brasilense* Sp7. Journal of Molecular Structure.

[53] Wang Y (2012). Differentiation in MALDI-TOF MS and FTIR spectra between two closely related species *Acidovorax oryzae* and *Acidovorax citrulli*. BMC Microbiology.

[54] Mohan CO, Gunasekaran S, Ravishankar CN (2019). Chitosan-capped gold nanoparticles for indicating temperature abuse in frozen stored products. npj Science of Food.

[55] Yee N, Benning LG, Phoenix VR, Ferris FG (2004). Characterization of metal-cyanobacteria sorption reactions: a combined macroscopic and infrared spectroscopic investigation. Environmental Science and Technology.

[56] Chakraborty M, Miao C, McDonald A, Chen S (2012). Concomitant extraction of bio–oil and value added polysaccharides from *Chlorella sorokiniana* using a unique sequential hydrothermal extraction technology. Fuel.

[57] Rianasari I (2016). Chemical template for synthesis of molecular sheets of calcium carbonate. Scientific reports.

[58] Gipson, K., Stevens, K., Brown, P., & Ballato, J. Infrared spectroscopic characterization of photoluminescent polymer nanocomposites. *Journal of Spectroscopy* (2015).

[59] Kim DS, Dhand V, Rhee KY, Park SJ (2015). Surface treatment and modification of graphene using organosilane and its thermal stability. Archives of Metallurgy and Materials.

[60] Pereira APDS (2017). Processing and characterization of PET composites reinforced with geopolymer concrete waste. Materials Research.

[61] Oliveira RN (2016). FTIR analysis and quantification of phenols and flavonoids of five commercially available plants extracts used in wound healing. Matéria (Rio de Janeiro).

[62] Costa S, Ferreira D, Ferreira A, Vaz F, Fangueiro R (2018). Multifunctional flax fibres based on the combined effect of silver and zinc oxide (Ag/ZnO) nanostructures. Nanomaterials.

[63] Sanches NB, Navarro S, Diniz MF, Dutra RDCL (2014). Characterization of additives typically employed in EPDM formulations by using FT-IR of gaseous pyrolyzates. Polímeros.

[64] Wang J, Zhang Q, Zhang Z, Li Z (2008). Antioxidant activity of sulfated polysaccharide fractions extracted from Laminaria japonica. International Journal of Biological Macromolecules.

[65] Qiao H, Wang G (2009). Effect of carbon source on growth and lipid accumulation in *Chlorella sorokiniana* GXNN01. Chinese Journal of Oceanology and Limnology.

[66] Pulz O, Gross W (2004). Valuable products from biotechnology of microalgae. Applied Microbiology and Biotechnology.

[67] Tannin-Spitz T, Bergman M, van-Moppes D, Grossman S, Arad SM (2005). Antioxidant activity of the polysaccharide of the red microalga *Porphyridium* sp. Journal of Applied Phycology.

[68] Duh P-D, Du P-C, Yen G-C (1999). Action of methanolic extract of mung bean hulls as inhibitors of lipid peroxidation and non-lipid oxidative damage. Food and Chemical Toxicology.

[69] Hussein M, AbdelGwad MA, Ragaa AA (2012). Bioactivity of *Ulva* spp. soluble polysaccharides on germination and growth of some crop plants. Mansoura Journal of Plant Protection and Pathology.

[70] Kumar VS (2004). Highly efficient Ag/C catalyst prepared by electro–chemical deposition method in controlling microorganisms in water. Journal of Molecular Catalysis A: Chemical.

[71] Alves A, Caridade SG, Mano JF, Sousa RA, Reis RL (2010). Extraction and physico–chemical characterization of a versatile biodegradable polysaccharide obtained from green algae. Carbohydrate Research.

[72] Mishra A, Kavita K, Jha B (2011). Characterization of extracellular polymeric substances produced by micro–algae *Dunaliella salina*. Carbohydrate Polymers.

[73] Skoog, D. A., Holler, F. J. & Nieman, T. A. Principles of Instrumental Analysis; Thomson Learning. Inc.: Toronto, ON (1998).

[74] Picout DR, Ross-Murphy SB (2003). Rheology of biopolymer solutions and gels. The Scientific World Journal.

[75] Bhatnagar M, Pareek S, Ganguly J, Bhatnagar A (2012). Rheology and composition of a multi-utility exopolymer from a desert borne cyanobacterium *Anabaena variabilis*. Journal of Applied Phycology.

[76] Freitas F (2009). Emulsifying behaviour and rheological properties of the extracellular polysaccharide produced by *Pseudomonas oleovorans* grown on glycerol byproduct. Carbohydrate Polymers.

[77] Khattar J (2010). Isolation and characterization of exopolysaccharides produced by the cyanobacterium *Limnothrix redekei* PUPCCC 116. Applied Biochemistry and Biotechnology.

[78] Sutherland, I. W. Biotechnology of microbial exopolysaccharides, Vol. 9. (Cambridge University Press, 1990).

[79] Phanjom P, Ahmed G (2015). Biosynthesis of silver nanoparticles by *Aspergillus oryzae* (MTCC No. 1846) and its characterizations. Nanoscience and Nanotechnology.

[80] El-Rafie H, El-Rafie M, Zahran M (2013). Green synthesis of silver nanoparticles using polysaccharides extracted from marine macro algae. Carbohydrate Polymers.

[81] Mohamedin A, El-Naggar NE, Shawqi Hamza S, Sherief A (2015). Green synthesis, characterization and antimicrobial activities of silver nanoparticles by *Streptomyces viridodiastaticus* SSHH-1 as a living nanofactory: Statistical optimization of process variables. Current Nanoscience.

[82] Morones JR (2005). The bactericidal effect of silver nanoparticles. Nanotechnology.

[83] El-Naggar, N. E. A., Mohamedin, A., Hamza, S. S., & Sherief, A. D. Extracellular biofabrication, characterization, and antimicrobial efficacy of silver nanoparticles loaded on cotton fabrics using newly isolated *Streptomyces* sp. SSHH-1E. *Journal of Nanomaterials* (2016).

[84] Senapati S, Ahmad A, Khan MI, Sastry M, Kumar R (2005). Extracellular biosynthesis of bimetallic Au–Ag alloy nanoparticles. Small.

[85] Chun Y-J, Shimada T, Waterman M, Guengerich F (2006). Understanding electron transport systems of *Streptomyces cytochrome* P450. Biochemical Society Transactions.

[86] Pandey S, Goswami GK, Nanda KK (2012). Green synthesis of biopolymer–silver nanoparticle nanocomposite: An optical sensor for ammonia detection. International Journal of Biological Macromolecules.

[87] Kanchana A, Agarwal I, Sunkar S, Nellore J, Namasivayam K (2011). Biogenic silver nanoparticles from *Spinacia oleracea* and *Lactuca sativa* and their potential antimicrobial activity. Digest Journal of Nanomaterials and Biostructures.

[88] El-Naggar NEA, Abdelwahed NA (2014). Application of statistical experimental design for optimization of silver nanoparticles biosynthesis by a nanofactory *Streptomyces viridochromogenes*. Journal of Microbiology.

[89] Magudapathy P, Gangopadhyay P, Panigrahi B, Nair K, Dhara S (2001). Electrical transport studies of Ag nanoclusters embedded in glass matrix. Physica B: Condensed Matter.

[90] Mukherjee P (2001). Fungus-mediated synthesis of silver nanoparticles and their immobilization in the mycelial matrix: a novel biological approach to nanoparticle synthesis. Nano Letters.

[91] Singh M, Singh S, Prasad S, Gambhir I (2008). Nanotechnology in medicine and antibacterial effect of silver nanoparticles. Digest Journal of Nanomaterials and Biostructures.

[92] Potts M (1999). Mechanisms of desiccation tolerance in cyanobacteria. European Journal of Phycology.

[93] Vázquez G, Moreno-Casasola P, Barrera O (1998). Interaction between algae and seed germination in tropical dune slack species: a facilitation process. Aquatic Botany.

[94] Wen-yu L (2008). Extraction of polysaccharide from *Nostoc flagelliform* and its effect on the germination rate of crop seeds. Journal of Anhui Agricultural Sciences.

[95] Osman MEH, El-Sheekh MM, El-Naggar AH, Gheda SF (2010). Effect of two species of cyanobacteria as biofertilizers on some metabolic activities, growth, and yield of pea plant. Biology and Fertility of Soils.

[96] Drazkiewicz, M. Chlorophyllase: occurrence, functions, mechanism of action, effects of external and internal factors (Review). *Photosynthetica* (Czech Republic) (1994).

[97] Stirk WA, Ördög V, Van Staden J (1999). Identification of the cytokinin isopentenyladenine in a strain of *Arthronema africanum* (Cyanobacteria). Journal of Phycology.

[98] Haroun S, Hussein M (2003). The promotive effect of algal biofertilizers on growth, protein pattern and some metabolic activities of *Lupinus termis* plants grown in siliceous soil. Asian Journal of Plant Sciences.

[99] Chen L, Li D, Liu Y (2003). Salt tolerance of *Microcoleus vaginatus* Gom., a cyanobacterium isolated from desert algal crust, was enhanced by exogenous carbohydrates. Journal of Arid Environments.

[100] Bograh A, Gingras Y, Tajmir-Riahi H, Carpentier R (1997). The effects of spermine and spermidine on the structure of photosystem II proteins in relation to inhibition of electron transport. FEBS Letters.

[101] Zheleva D, Tsonev T, Sergiev I, Karanov E (1994). Protective effect of exogenous polyamines against atrazine in pea plants. Journal of Plant Growth Regulation.

[102] Mohiuddin M, Das A, Ghosh D (2000). Growth and productivity of wheat as influenced by integrated use of chemical fertilizer, biofertilizer and growth regulator. Indian Journal of Plant Physiology.

[103] Kavipriya, R., Dhanalakshmi, P., Jayashree, S. & Thangaraju, N. Seaweed extract as a biostimulant for legume crop, green gram. *Journal of Ecobiotechnology* **3** (2011).

[104] Thirumaran G, Arumugam M, Arumugam R, Anantharaman P (2009). Effect of seaweed liquid fertilizer on growth and pigment concentration of *Abelmoschus esculentus* (I) Medikus. *American-Eurasian*. Journal of Agronomy.

[105] Stainer RY, Kunisawa R, Mandel M, Cohen-Bazire G (1971). Purification and properties of unicellular blue-green algae (Order Chroococcales). Bacteriological Reviews.

[106] Hoshaw, R. & Rosowski, J. Methods for microscopic algae. Handbook of Phycological Methods. Cambridge University Press, New York, 53–67 (1973).

[107] Philipose, M. Chlorococcales, Vol. 8. (Indian Council of Agricultural Research, 1967).

[108] Wang H, Wang Q, Wang S, Wang Z, Shen J (2006). Extraction, isolation and structure identification of polysaccharide in root of *Salvia miltiorrhiza*. Zhongguo Zhong Yao Za Zhi = China journal of Chinese Material Medica.

[109] Dubois M, Gilles KA, Hamilton JK, Rebers P, Smith F (1956). Colorimetric method for determination of sugars and related substances. Analytical Chemistry.

[110] Chen M, Wu WW, Nanz D, Sticher O, Leonticins DH (1997). Five triterpene saponins from *Leontice kiangnanensis*. Phytochemistry.

[111] Wang Y (2004). Chemical components and molecular mass of six polysaccharides isolated from the sclerotium of Poriacocos. Carbohydrate Research.

[112] Lowry OH, Rosebrough NJ, Farr AL, Randall RJ (1951). Protein measurement with the Folin phenol reagent. The Journal of Biological Chemistry.

[113] Qiao D (2009). Antioxidant activities of polysaccharides from *Hyriopsis cumingii*. Carbohydrate polymers.

[114] Fernandes H, Lupi F, Tomé M, Sá-Correia I, Novais J (1991). Rheological behaviour of the culture medium during growth of the microalga *Botryococcus braunii*. Bioresource Technology.

[115] Seyam A-FM, Hudson SM, Ibrahim HM, Waly AI, Abou-Zeid NY (2012). Healing performance of wound dressing from cyanoethyl chitosan electrospunfibres. Indian Journal of Fibre and Textile Research.

[116] Elarroussia H (2016). Microalgae polysaccharides a promising plant growth biostimulant. Journal of Biomass Utln.

[117] Hernández-Herrera RM, Santacruz-Ruvalcaba F, Zañudo-Hernández J, Hernández-Carmona G (2016). Activity of seaweed extracts and polysaccharide-enriched extracts from *Ulva lactuca* and *Padina gymnospora* as growth promoters of tomato and mung bean plants. Journal of applied phycology.

[118] Quarrie S, Jones H (1979). Genotypic variation in leaf water potential, stomatal conductance and abscisic acid concentration in spring wheat subjected to artificial drought stress. Annals of Botany.

[119] Vermaat J (1995). Meadow maintenance, growth and productivity of a mixed Philippine seagrass bed. Marine Ecology Progress Series.

[120] Metzner H, Rau H, Senger H (1965). Untersuchungen zur synchronisierbarkeit einzelner pigmentmangel–mutanten von *Chlorella*. Planta.

[121] Beers RF, Sizer IW (1952). A spectrophotometric method for measuring the breakdown of hydrogen peroxide by catalase. Journal of Biological Chemistry.

[122] Hakiman M, Maziah M (2009). Non enzymatic and enzymatic antioxidant activities in aqueous extract of different Ficus deltoidea accessions. Journal of Medicinal Plants Research.

[123] Gillespie KM, Chae JM, Ainsworth EA (2007). Rapid measurement of total antioxidant capacity in plants. Nature Protocols.

[124] Zobel RW, Wright MJ, Gauch HG (1988). Statistical analysis of a yield trial. Agronomy Journal.

